# Distribution of SARS-CoV-2 Lineages in the Czech Republic, Analysis of Data from the First Year of the Pandemic

**DOI:** 10.3390/microorganisms9081671

**Published:** 2021-08-05

**Authors:** Petr Klempt, Ondřej Brzoň, Martin Kašný, Kateřina Kvapilová, Petr Hubáček, Aleš Briksi, Matěj Bezdíček, Vladimira Koudeláková, Martina Lengerová, Marian Hajdúch, Pavel Dřevínek, Šárka Pospíšilová, Eva Kriegová, Milan Macek, Petr Kvapil

**Affiliations:** 1 Institute of Applied Biotechnologies, Služeb 3056/4, 108 00 Prague, Czech Republic; brzon@iabio.eu (O.B.); kasny@iabio.eu (M.K.); kvapilova@iabio.eu (K.K.); kvapil@iabio.eu (P.K.); 2 Department of Parasitology, BIOCEV, Faculty of Science, Charles University, Průmyslová 595, 252 50 Vestec, Czech Republic; 3 Department of Genetics and Microbiology, Faculty of Science, Charles University, Albertov 6, 128 00 Prague, Czech Republic; 4 Department of Medical Microbiology, 2nd Faculty of Medicine, Charles University and Motol University Hospital, V Úvalu 84, 150 06 Prague, Czech Republic; petr.hubacek@fnmotol.cz (P.H.); ales.briksi@fnmotol.cz (A.B.); pavel.drevinek@lfmotol.cuni.cz (P.D.); 5 Center of Molecular Biology and Genetics, Department of Internal Medicine-Haematology and Oncology, University Hospital Brno and Faculty of Medicine, Masaryk University, Černopolní 212/9, 625 00 Brno, Czech Republic; bezdicek.matej@fnbrno.cz (M.B.); lengerova.martina@fnbrno.cz (M.L.); pospisilova.sarka@fnbrno.cz (Š.P.); 6 Laboratory of Experimental Medicine, Institute of Molecular and Translational Medicine, Faculty of Medicine and Dentistry, Palacky University Olomouc and University Hospital Olomouc, Hněvotínská 5, 77 515 Olomouc, Czech Republic; vladimira.koudelakova@upol.cz (V.K.); marian.hajduch@upol.cz (M.H.); 7 Department of Immunology, Faculty of Medicine and Dentistry, Palacký University Olomouc and University Hospital, Hněvotínská 3, 775 15 Olomouc, Czech Republic; eva.kriegova@fnol.cz; 8 Department of Biology and Medical Genetics, 2nd Faculty of Medicine-Charles University and Motol University Hospital, V Úvalu 84, 150 05 Prague, Czech Republic; milan.macek.jr@lfmotol.cuni.cz

**Keywords:** SARS-CoV-2, metagenomics, variants, phylogeny, massively parallel sequencing

## Abstract

In the Czech Republic, the current pandemic led to over 1.67 million SARS-CoV-2- positive cases since the recording of the first case on 1 March 2020. SARS-CoV-2 genome analysis is an important tool for effective real-time quantitative PCR (RT-qPCR) diagnostics, epidemiology monitoring, as well as vaccination strategy. To date, there is no comprehensive report on the distribution of SARS-CoV-2 genome variants in either the Czech Republic, including Central and Eastern Europe in general, during the first year of pandemic. In this study, we have analysed a representative cohort of SARS-CoV-2 genomes from 229 nasopharyngeal swabs of COVID-19 positive patients collected between March 2020 and February 2021 using validated reference-based sequencing workflow. We document the changing frequency of dominant variants of SARS-CoV-2 (from B.1 -> B.1.1.266 -> B.1.258 -> B.1.1.7) throughout the first year of the pandemic and list specific variants that could impact the diagnostic efficiency RT-qPCR assays. Moreover, our reference-based workflow provided evidence of superinfection in several samples, which may have contributed to one of the highest per capita numbers of COVID-19 cases and deaths during the first year of the pandemic in the Czech Republic.

## 1. Introduction

The global spread of novel coronavirus SARS-CoV-2 has been associated with a natural evolution of its genome. Since the reporting of the first case in Wuhan (China) in December 2019 [1], a large number of sequence variants has been described [2,3]. Following the emergence of new variants, the constant need for updates of variant nomenclature [4,5] has been reflected by the GISAID nomenclature system [2] and the Nextstrain nomenclature system [3], including a dynamic virus nomenclature system which reflects the spread of the SARS-CoV-2 [6]. We decided to use the latest version of nomenclature as reported elsewhere [6].

**Figure 1:**
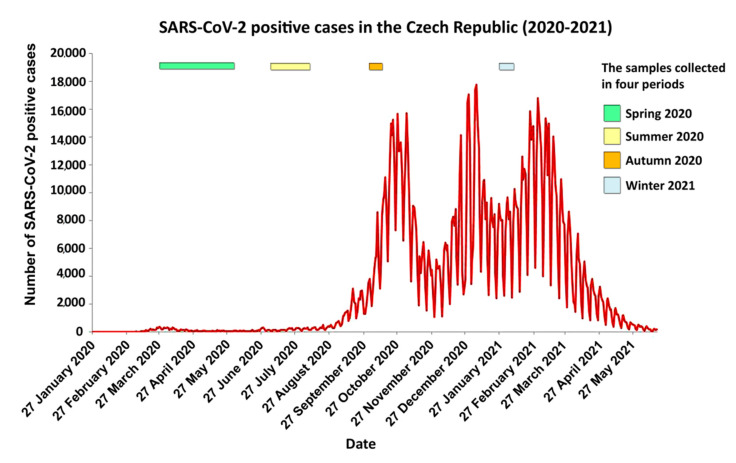
SARS-CoV-2 positive cases in the Czech Republic. Graph based on a data source available at https://onemocneni-aktualne.mzcr.cz/api/v2/covid-19 (accessed on 18 June 2021). Coloured bars in the upper part of the graph illustrate sample collection time range for each of the analysed cohorts (Spring 2020, Summer 2020, Autumn 2020, and Winter 2021).

The first three cases of COVID-19 positive patients in the Czech Republic, a country with a population of approximately 10 million, were reported on 1 March 2020 in connection with people returning from winter holidays in northern Italy in late February [7]. Since then, our country experienced several waves of the pandemic (Figure 1) and over 1.67 million of SARS-CoV-2 positive cases have been confirmed in the country as of July 2021 [8].

The second wave of rapid growth of SARS-CoV-2 positive cases in the Czech Republic occurred in September–October 2020 (Figure 1) [8]. This wave had one of the highest ‘positivity ratios’ in Europe, with positivity found in over 30% of submitted RT-qPCR tests [9,10]. The following third wave in December 2020 surpassed the September–October wave in terms of daily incidence of positive cases (Figure 1) [8]. Both of these waves of positive cases, i.e., the September–October and December 2020 wave (Figure 1), tended to be associated with a relaxation of COVID-19 restrictions adopted by the central government and with national elections [8]. The last wave, which culminated on 2 March 2021, was associated with the spread of the ‘Alpha (British)’ variant B.1.1.7 [11]. We thus discuss our findings also in relation to local specifics of the COVID-19 pandemic.

Generally speaking, new genetic variants of SARS-CoV-2 constantly emerge and start circulating around the world. Recently, based on their predicted impact on transmission, diagnosis, therapy, or immune escape, SARS-CoV-2 variants have been divided into three classes: (i) variants of interest (VOI), (ii) variants of concern (VOC), and (iii) variants of severe consequence (VOHC) [12]. Current variants of interest (VOI) are B.1.1.7 (currently termed Alpha), B.1.351 (Beta), B.1.617.2 (Delta), and P.1 (Gamma). VOCs include variants B.1.427/9 (Epsilon), B.1.525 (Eta), B.1.526 (Iota), B.1.617.1 (Kappa); variants in this group require special consideration in terms of diagnosis, treatment, and vaccination. There are currently no SARS-CoV-2 variants belonging to the group of VOHCs.

The main aim of this study was to obtain and provide high-quality full-length SARS-CoV-2 genome sequences that reflect the local and community spread of SARS-CoV-2 in the Czech Republic, which led to one of the highest ‘positivity ratios’ and unfortunately also one of the highest per capita SARS-CoV-2-related deaths during the first year of the pandemic in Europe [9,10].

We decided to work with a validated workflow because this method not only provides information about the SARS-CoV-2 genome variant but also enables detection of superinfections (two different virus variants in the same sample). A comparison of sequenced variants from the first year of the pandemic in the Czech Republic has not yet been undertaken, nor is there any comprehensive report on the distribution of SARS-CoV-2 genome variants in other Central and Eastern European countries.

For the purpose of this research, we introduce two validated sequencing analysis methods of detection of SARS-CoV-2 variants. We also discuss the potential of each of these methods to discover possible superinfection. Next, we performed SARS-CoV-2 phylogenetic analysis using consensus sequence from reference mapping. Our results indicate changes in the pattern of SARS-CoV-2 variants circulating in the Czech Republic and show trends similar to other European countries. Finally, we evaluate the impact of described variants on the protocols for RT-qPCR diagnostics of SARS-CoV-2 [13].

## 2. Materials and Methods

### 2.1. Samples

In total, we received 229 samples of nasopharyngeal swabs from patients positively diagnosed with COVID-19 via our partnership with large Czech hospitals (Table 1), which represent key catchment areas within the Czech Republic (metropolitan areas of Prague, Brno, Pilsen, Kladno, and Olomouc). For collection dates and locations, see Supplementary Table S1. Isolation and further processing of the SARS-CoV-2 samples were performed according to a procedure used in our previous study [14,15].

### 2.2. Generation of Controls

Negative and positive RNA controls were prepared as reported previously [12]. In short, for generation of negative controls (nc), we used 5 ng (nc) of human breast tumour RNA (HBT) (Takara Bio; Saint-Germain-en-Laye, France). Then we prepared two positive controls (pc) from 5 ng of HBT spiked by two synthetic SARS-CoV-2 RNA controls (SARS-CoV-2 RNA control 1 [Australia/VIC01/202, GenBank ID: MT007544.1] and SARS-CoV-2 RNA control 2 [Wuhan-Hu-1, GenBank ID: MN908947.3], Twist Bioscience, San Francisco, CA, USA) in a ratio of 1:1, with the aim of obtaining the positive control with 1000 + 1000 copies of each.

### 2.3. Calculation of Ct for Samples and Controls

The number of virus copies in each sample was calculated according to Ct values measured by adoption of direct SARS-CoV-2 RT-qPCR assay for synthetic RNA positive controls diluted to a defined viral copy concentration [15].

### 2.4. NGS Library Preparation

Overall, NGS libraries were prepared from 229 isolates in sets specific for each solution of library preparation. Thirteen samples were prepared using NEB+TWIST and Illumina workflows according to the manufacturer’s instructions.

#### 2.4.1. NGS Library Preparation—Illumina

A set of 126 isolates (Ct range 11.29–26.39; see Table S1) and one positive (pc-Illumina) and one negative (nc-Illumina) control were transcribed into ds cDNA using NEBNext^®^ RNA First Strand Synthesis module and NEBNext UltraII Directional RNA Second Strand Synthesis module (New England Biolabs; Ipswich, MA, USA) following the manufacturer’s protocol.

Libraries were prepared using Nextera Flex for Enrichment (pre-enrichment part of manufacturer’s protocol; https://emea.support.illumina.com/sequencing/sequencing_kits/illumina-dna-prep-with-enrichment/documentation.html (accessed on 30 July 2021)). Next, libraries were combined in twelve plexes by 7–11 samples (including controls) based on the Ct values (in order to minimise the Ct difference in samples within each plex) and enriched using the Respiratory Virus Oligo Panel (Illumina, San Diego, CA, USA), following the manufacturer’s protocol (https://www.illumina.com/content/dam/illumina-marketing/documents/products/appnotes/coronavirus-enrichment-product-list-1270-2020-004.pdf accessed on 30 July 2021).

Enriched plexes were equally pooled based on evaluation by Qubit 2.0 and Bioanalyzer 2100 and sequenced on the MiSeq platform in a run with configuration 2 × 176 bp using MiSeq Reagent Kit v3 (600 cycle) (Illumina, San Diego, CA, USA).

#### 2.4.2. NGS Library Preparation: NEBNext + Twist (NEB + TWIST) Combined Workflow

Libraries were prepared from 28 isolates (Ct range 11.29–23.29; see Table S1) and positive (pc-NEB + TWIST) and negative (nc-NEB + TWIST) controls using the NEBNext Ultra II Directional RNA Library Prep Kit from Illumina (New England Biolabs; Ipswich, MA, USA). Libraries were combined in 4 plexes from 6–8 samples in each (including controls) based on the Ct values. Subsequent enrichment reaction was performed using the SARS-CoV-2 Research Panel (Twist Bioscience, San Francisco, CA, USA) following the Twist Target Enrichment Workflow protocol (https://www.twistbioscience.com/resources/protocol/twist-target-enrichment-protocol-use-twist-ngs-workflow accessed on 14 May 2021). Enrichment plexes were pooled and sequenced on the MiSeq platform (Illumina; San Diego, CA, USA) using MiSeq Reagent Kit v3 (600 cycles), MiSeq Reagent V2, and MiSeq Reagent V2 Nano (500 cycles both) in the 2 × 300 bp and 2 × 250 bp sequencing run configuration, respectively.

#### 2.4.3. NGS Library Preparation-Twist Workflow

The third set of 93 samples (Ct range 6.43–23.66; see Table S1) and positive (pc-TWIST) and negative (nc-TWIST) controls were prepared according to a protocol developed by Twist Bioscience (San Francisco, CA, USA) using Twist Library Preparation kits and target enrichment with SARS-CoV-2 Research panel (Twist Bioscience; San Francisco, CA, USA). The first part of the protocol (ds cDNA synthesis) relies on the ProtoScript II First Strand cDNA Synthesis Kit followed by NEBNext Ultra II Non-Directional RNA Second Strand Synthesis module (New England Biolabs; Ipswich, MA, USA). The protocol is available at: https://www.twistbioscience.com/resources/protocol/sars-cov-2-ngs-assay-ruo-protocol (accessed on 30 July 2021). Pool of prepared libraries were sequenced in 2 × 150 bp run on the NovaSeq 6000 platform (Illumina, San Diego, CA, USA).

### 2.5. Massively Parallel Sequencing

Libraries from each preparation were pooled based on a quality control evaluation. Further, libraries for MiSeq (prepared as described in 2.4.1 and 2.4.2) or NovaSeq 6000 (samples prepared as described in 2.4.3) were diluted and denatured according to the MiSeq or NovaSeq Denature and Dilute Guides (February 2019, v10 version and November 2020, v03 version).

### 2.6. Reference Mapping Data Analysis

CLC Genomics Workbench 20.0.4 (QIAGEN Gmbh; Hilden, Germany) bioinformatics workflow, Identify QIAseq SARS-CoV-2 Low Frequency and Shared Variants (Illumina) (0.1) were used for raw reads trimming, quality control, and reference mapping with default settings. Trimmed reads were mapped to the Wuhan SARS-CoV-2 reference genome (NC_045512). Where possible, data from the same biological samples prepared using the two library preparation methods were merged after QC (18 samples, Table S1). To detect potential superinfections, we disabled the fixed ploidy option and set the minimum variant frequency to 30% with at least 30X coverage and minimum average base quality of 20. Further information about the prepared workflow can be found on QIAGEN webpages (https://digitalinsights.qiagen.com/news/blog/discovery/building-workflows-for-SARS-CoV-2-mutation-analysis-in-qiagen-clc-genomics-workbench/ accessed on 30 July 2021).

Consensus sequences were extracted and aligned using the Create Alignment (1.02) module in CLC with default settings. Refined multiple alignment was analysed in IQ-Tree software (2.1.1) with GTR+F substitution model and 10,000 Ultrafast Bootstrap replicates [16]. Final phylogenetic tree was generated and edited in iTOL [17].

### 2.7. De Novo Assembly Data Analysis

Low quality and/or short reads and adaptors were trimmed using the CLC Genomic Workbench module Trim Reads (2.4) with default settings. De novo assembly module (1.5) was subsequently used with automatic word size, mismatch cost 2, insertion and deletion cost 3. Contigs over 20 kb were compared with consensus sequences from reference mapping of the corresponding biological sample.

### 2.8. SARS-CoV-2 Lineage Classification

All SARS-CoV-2 genomic sequences were uploaded to the Pangolin web service to assign the most likely SARS-CoV-2 lineage to our samples (Pango nomenclature) [6].

### 2.9. Data Availability

Consensus FASTA files from all studied viral genomes were uploaded to the Global Initiative on Sharing All Influenza Data (GISAID) [2].

### 2.10. Ethics

All analyses were performed in accordance with ethical standards of the institutional and/or national research committee and respected the 1964 Helsinki Declaration and its later amendments or comparable relevant ethical standards. Anonymised human nasopharyngeal swab samples were obtained in the context of a COVID-19 monitoring study at Palacký University Olomouc, University Hospital Olomouc, University Hospital Brno, Regional Hospital in Kladno, Motol University Hospital Prague, and University Hospital Pilsen.

## 3. Results and Discussion

One of the purposes of this study was to provide information about the distribution of SARS-CoV-2 genome variants within isolates collected during the first year (March 2020–February 2021) of COVID-19 pandemic in the Czech Republic. In comparison to neighbouring countries, namely Austria [18], Poland [19], or Germany (https://www.rki.de/ accessed on 24 July 2021), the Czech Republic developed no sustained countrywide effort focused on the sequencing of SARS-CoV-2-positive samples during the first year of the pandemic. For the purpose of our study, we have adopted formerly tested library preparation solutions for massive parallel sequencing [14] of isolates of nucleic acids from nasopharyngeal swabs of SARS-CoV-2-positive patients collected since early spring 2020 (March 2020) until February 2021 [20,21,22] in diagnostic laboratories located in five major hospitals (see Materials and Methods) selected to represent the progress of the pandemic within the entire country. The analysed samples reflect four waves of the pandemic: Spring 2020 (March–June 2020), Summer 2020 (July–September 2020), Autumn 2020 (October 2020), and Winter 2021 (January–February 2021; Figure 1).

**Figure 2:**
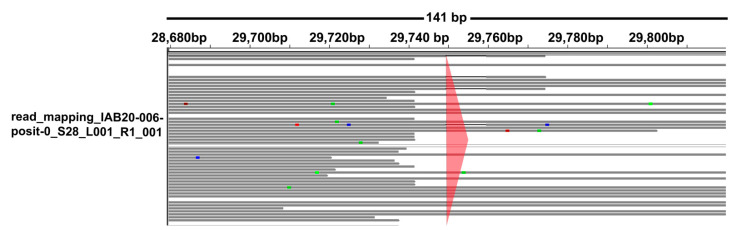
Variants on control samples using the Integrative Genomics Viewer. Ten nucleotide long deletion in position 29,749 of the positive control was not successfully called in workflows using the Twist SARS-CoV-2 panel due to its location at the end of the genomic sequence. The observed decrease of coverage in this region is due to the performance of the Twist panel, which does not directly target this genomic region. In our control design, where variant should be present in approximately 50% of reads, it resulted in a stable decrease of coverage.

Using enrichment workflows (Illumina Respiratory Virus Oligo panel and the Twist SARS-CoV-2 Research Panel; see Material and Methods), we obtained high quality sequencing data required for viral whole-genome analysis of 229 samples from across the country. To analyse the generated sequencing data, we tested two approaches for data assembly: (a) reference-based mapping, which results in a consensus call, and (b) a *de novo* assembly method. Primarily, we tested the reference-based mapping methods on data from our synthetic controls to validate our data analysis workflow in a CLC Genomics Workbench tool (v.20.0.4; QIAGEN GmbH; Hilden, Germany). The SARS-CoV-2 positive control generated from a mixture of Australia/VIC01/202 and Wuhan-Hu-1 synthetic controls (2000 copies per reaction in total; see Material and Methods) enabled us to assess our ability to detect a possible superinfection. Using this approach, we were able to reliably identify all three SNPs (positions 19065, 22303, and 26144 based on the MN908947.3), which distinguish the Wuhan-Hu-1 (MN908947.3) genome variant from the variant detected in Australia/VIC01/202 (MT007544.1) (Table S2). The fourth variant (deletion of 10 nucleotides in position 29749) was successfully identified only in a positive control processed using the Illumina panel. The positive control enriched using Twist panel did not have sufficient coverage in the genomic regions due to the location and the end of genomic sequence. In our control design, where variant should be present in ~50% of reads, it resulted in a stable decrease rather than a significant drop in coverage (Figure 2). This phenomenon could potentially influence ability to successfully recall all indels at the beginning and the end of the SARS-CoV-2 genome in case of superinfection. Importantly, in our samples with potential superinfection prepared by both library preparations, all variants correspond.

Once we confirmed that the data analysis pipeline is successful, we applied it to massively parallel sequencing of data from all samples. In total, all published sequences of 229 samples met the following criteria for subsequent data analysis: near-full genome sequence covered in range of 56 to 29,797 bp positions, with no ‘N positions’ and minimal coverage of 30× per variant call.

To validate the reference mapping approach, we decided to compare identified variants with variants detected using the de novo assembly approach. Comparisons were performed using 51 de novo assemblies that passed the same criteria for data analysis as those used for reference mapping (see above). Both sets of identified variants classified samples into identical SARS-CoV-2 lineages. The de novo assembly approach did not, however, perform well on our control samples (mixture of two SARS-CoV-2 genome variants) and identified only one variant in the control (Table S2). De novo assembly pipeline was also not successful in samples where the reference mapping approach identified the presence of two or more variants (potential superinfection) (Table S2). We have therefore decided to continue using only the reference mapping approach as described above.

**Figure 3:**
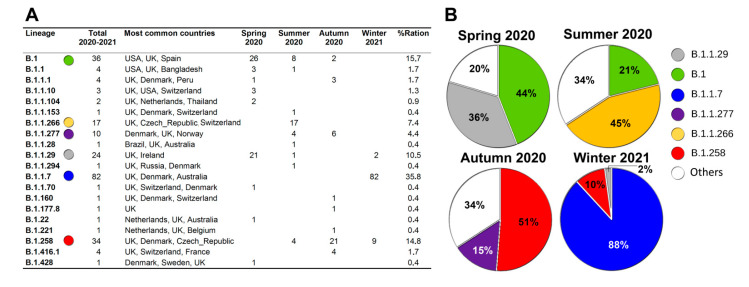
SARS-CoV-2 lineages (**A**) recorded for samples collected in four periods (**B**) between March 2020 and February 2021. Pangolin web service was used to assign SARS-CoV-2 lineages to our samples (https://cov-lineages.org accessed on 14 March 2021).

Our data analysis pipeline classified samples based on their sequence using the Pangolin web services tool [6]. The variants from 229 samples clustered in 20 SARS-CoV-2 lineages (see Table S1 and Figure 3), whereby most samples fell within four dominant lineages with ~10–36% of the total for B.1, B.1.1.29, B.1.258, and B.1.1.7 each (Figure 3).

The B.1 lineage, at the time detected mainly in the USA, United Kingdom, and Spain, was the dominant lineage (>44% of all samples) in the Spring 2020 collection (March–June 2020). The second most prevalent lineage during this period was B.1.1.29, frequently found especially in the United Kingdom and Ireland, which accounted for over 35% of all collected samples. In total, samples collected during Spring 2020 showed the presence of nine lineages.

The period of Summer 2020 (samples collected in July–September 2020) again showed the presence of nine different SARS-CoV-2 lineages. The two lineages with highest prevalence in the Spring 2020 collection (B.1 and B1.1.29) were still present (in 21% and 2.6% of samples, respectively) but the dominant position was now assumed by the B.1.1.266 lineage, found especially in the United Kingdom, Czech Republic, and Switzerland, which was present in 44.7% of all samples, followed by the B.1. (21%), B.1.258 (United Kingdom, Denmark, and the Czech Republic) and B.1.1.277 (Denmark, United Kingdom, and Norway) lineages with a share of 10.5% each.

In the Autumn 2020 collection (samples collected on 2–6 October 2020, Table S1), the B.1.258 (represented mainly in the United Kingdom, Denmark, and the Czech Republic) became the dominant lineage with overall share of 51%. The second most represented lineage during this period was B.1.1.277 (prevalent especially in Denmark, United Kingdom, and Norway) with presence in 14.6% of samples. The B.1. lineage, which was dominant during the Spring 2020 period, was detected only in two cases and in the Winter 2021 sample collection was absent completely. It is questionable whether the ‘dramatic’ pandemic situation in the autumn of 2020 in the Czech Republic [9,10] was driven by changes in the distribution of lineages (e.g., high representation of the B.1.258 lineage), related to the specifics of local situation characterised by arbitrarily relaxed COVID-19 governmental restrictions (e.g., return of pupils to schools from 1 September until national elections were held on 2–3 October 2020), or–and most likely–by a combination of both factors.

**Figure 4:**
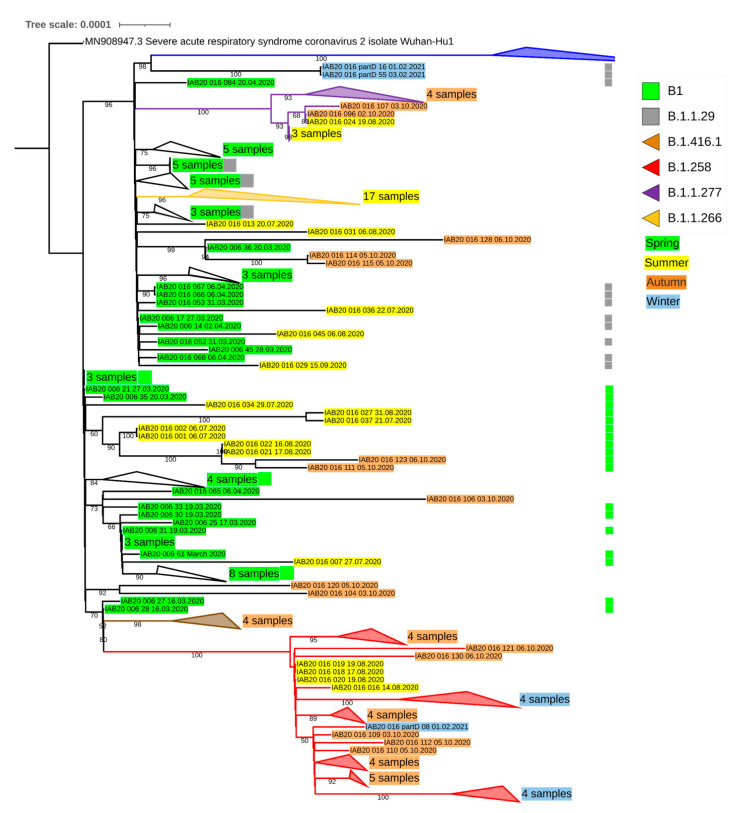
A phylogenetic tree based on SARS-CoV-2 sequences showing the particular lineages recorded for samples collected during four periods between March 2020 and February 2021. For better orientation, the tree is collapsed. For the full concatenated tree, see Supplementary Materials Figure S1. Consensus sequences were extracted and aligned using the Create Alignment (1.02) module in CLC with default settings. Refined multiple alignment was analysed in IQ-Tree software (2.1.1) with GTR + F substitution model and 10,000 Ultrafast Bootstrap replicates. Parameters used for constructing the phylogenetic tree are also mentioned in Material and Methods.

The B.1.258 lineage was the second most abundant (9.7%) in samples collected in Winter 2021 (28 January–5 February 2021, Table S1) but the most abundantly represented lineage during this collection was the ‘Alpha’ B.1.1.7 lineage (>88%) (Figure 3 and Figure 4 and Figure S1). This distant lineage, described in a subset of samples collected on 20–21 September 2020 in South East England [11] was not identified in our Summer–Autumn 2020 datasets (June–October 2020) but was clearly present in samples collected from the beginning of 2021. Similar to the pandemic situation in autumn 2020, active spread of variants, including the B.1.1.7, during winter 2020–2021 may have been enabled by a repeated relaxation of pandemic regulations adopted by the Czech government shortly before the Christmas 2020 holiday season.

It should be noted that the last collection of samples (Winter 2021) was selected in part based on epidemiological findings, such as COVID-19 symptom severity, positivity after first or second dose of vaccine, or, in most cases, an unusual profile of qPCR traces. In general, the gradual elimination of most lineages from our record concurs with what was previously described based on a significantly higher set of samples in the United Kingdom [23].

Interestingly, five samples from the Spring 2020 period, six samples from the Summer 2020 period, four samples from the Autumn 2020 period, and eight samples from the Winter period 2021 showed the presence of two or more variants at the same position, indicating a potential superinfection event (Table S2). Table S2 shows the support for all positions with two variants in the same position. To eliminate the library preparation bias, samples prepared by both Illumina and Twist methods (IAB20_006_07, IAB20_006_10, IAB20_006_15) were analysed separately. In all three cases, the Illumina and Twist approach identified the same viral genome variants. Samples successfully assembled using the de novo approach contained only variants with a higher frequency in the corresponding position (Table S2). This finding raises the question about the limitations of *de novo* assembly approach regarding the detection of eventual superinfection events. Recently, there emerged several reports of superinfection with two strains of SARS-CoV-2 [24,25,26]. Our sequencing data showed that ~10% of the samples examined (23 of 229 samples) had two SARS-CoV-2 variants present, with a similar frequency (12.8%) observed in an independent Iranian cohort [25]. Whether superinfection contributed to one of the highest rates of COVID-19 and the high number of COVID-related deaths during the first year of the pandemic in the Czech Republic remains unknown.

Another important goal of this project was to evaluate the impact of variants circulating in our population on the accuracy of routine diagnostic methods. Looking at regions of primer annealing for RT-PCR assays of SARS-CoV-2 [13], we detected four variants in Spring 2020, eleven variants in the Summer 2020 and Autumn 2020, and seven variants in the Winter 2021 samples (Table S3). When assessing the critical sites recommended by WHO protocols, our dataset showed that the variant at position 26700 is present at 5′ of the forward primer for the E gene (E_Sarbeco_F, primer 26269-26294) [27], while the variant at position 14111 might hinder the RdRp probe (nCoV IP4, probe 14105-14123), thereby potentially affecting the diagnostic accuracy of RT-qPCR-based assays, which were used for the detection of SARS-CoV-2 in the Czech Republic.

The present study has several limitations in terms of interpretation of evidence of superinfection we found in ~10% of analysed samples. Due to the specific features of SARS-CoV-2 RT-qPCR diagnostics [28] related to extremely high diversity of viral copies in individual samples and a vast number of analysed samples in diagnostic laboratories, contamination of samples cannot be completely excluded. On the other hand, all samples came from accredited and experienced diagnostic laboratories with established laboratory quality control systems (often ISO15189:2013; data available upon request), which ought to minimise the likelihood of accidental contamination. Another limitation is that all samples were anonymous and no follow-up samples or clinical data on the course of COVID-19 were thus available to us. Furthermore, as shown elsewhere [24], repeated analysis of isolates from several collections (independent, separated isolations from several timepoints) should be investigated in future studies. Authors of the present study are aware of this; however, consider the presented approach being capable to discriminate eventual superinfection to be beneficial.

The current experience with SARS-CoV-2 sequencing from the first year of pandemic further supports the need for diagnostic sequencing to control the spread of the virus in the future. Together with epidemiological data, sequencing can help authorities rapidly deploy additional support, such as bump testing, increased contact tracing, or isolation measures, in areas where known or new VOCs will occur.

## 4. Conclusions

To the best of our knowledge, this study offers the first comprehensive report of SARS-CoV-2 genome variant distribution in a Central European country. This report summarises the effort to monitor the distribution of SARS-CoV-2 genome variants in the Czech Republic from March 2020 to February 2021, thus substantiating the relevance of viral whole genome sequencing with respect to the natural evolution of SARS-CoV-2 during the first year of COVID-19 pandemic in the Czech Republic. Using a validated hybridisation-capture workflow, we gathered and published in the GISAID database high-quality whole-genome data from 229 representative samples.

Our data provide evidence of the changing frequency of dominant variants (from B.1 -> B.1.1.266 -> B.1.258 -> B.1.1.7) during the sampled periods covering the spring, summer, autumn of 2020, and winter 2021 i.e., during the first year of the pandemic in the Czech Republic. A comparison between de novo assembly pipeline approach and our reference-based workflow highlighted differences in the robustness of these methods with respect to detecting superinfection events. While our reference-based workflow, which was validated using mixed positive controls, provided evidence of superinfection in several samples, the de novo assembly pipeline turned out to be unable to identify superinfection events either in the positive controls or in the clinical samples. Finally, we list the variants which might impact the diagnostic efficiency of RT-qPCR assays.

## Figures and Tables

**Table 1:** The number of samples collected each month of four periods.

Period	Month	Number of Samples	Number of Samples/Period
	March	41	
Spring 2020	April	15	59
	June	3	
	July	13	
Summer 2020	August	24	38
	September	1	
Autumn 2020	October	39	39
Winter 2021	January	6	93
	February	87	
Total			229

## Data Availability

The same standards for ethics, copyright, attributions, and permissions apply to the Supplementary Materials and the body of the article. Supplementary Materials are not edited by Multidisciplinary Digital Publishing Institute and the journal is not responsible for the maintenance of any links or email addresses provided therein.

## Supplementary Materials

The following are available online at https://www.mdpi.com/article/10.3390/microorganisms9081671/s1. Table S1. Samples collected during four periods spanning from March 2020 to February 2021 and processed using the Illumina, NEB + TWIST and TWIST, and NGS library preparation approach. Lineage, samples assigned to particular SARS-CoV-2 lineages (Pangolin web service); collection place, samples obtained from Czech hospitals from five cities; Ct, reflecting the viral load. Table S2. Results of the reference mapping approach to the detection of SARS-CoV-2 genome variants. Different SARS-CoV-2 genome variants detected by reference mapping in the same position in positive controls and in the samples evaluated as potentially superinfected. Library preparation, samples processed using the Illumina, NEB+TWIST and TWIST, and NGS library preparation approach; position, variant position on the SARS-CoV-2 Wuhan-Hu-1 reference genomics sequence.; variant allele frequency; coverage, the number of reads covering a given position.; reference, allele of the reference genome; variant allele, variant position that differs from the reference; de novo assembly, column showing whether the sample was assembled also de novo or not; results, De Novo, results of variant calling over de novo assembled genomes. Table S3. Mutations detected in the SARS-CoV-2 genome (NC_045512) corresponding to the regions of primer/probe annealing for RT-qPCR assays. Gene, name of the gene in which the mutation was detected; Position, the exact position of mutation; Primers/Probes, the range related to SARS-CoV-2 genome defined for primer/probe design for RT-qPCR. Figure S1. A phylogenetic tree based on SARS-CoV-2 sequences showing the particular lineages recorded for samples collected at four collection points during from March 2020 until February 2021. Full concatenated tree. Consensus sequences were extracted and aligned using the Create Alignment (1.02) module in CLC with default settings. Refined multiple alignment was analysed in the IQ-Tree software (2.1.1) with GTR + F substitution model and 10,000 Ultrafast Bootstrap replicates. All legends to the Supplementary Figures and Tables are presented in the supplementary files.
