# Supplementary figures and images for: Distribution of SARS-CoV-2 Lineages in the Czech Republic, Analysis of Data from the First Year of the Pandemic
Source: Microorganisms. 2021 Aug 5;9(8):1671. doi: 10.3390/microorganisms9081671 (PMC8397935; doi:10.3390/microorganisms9081671)

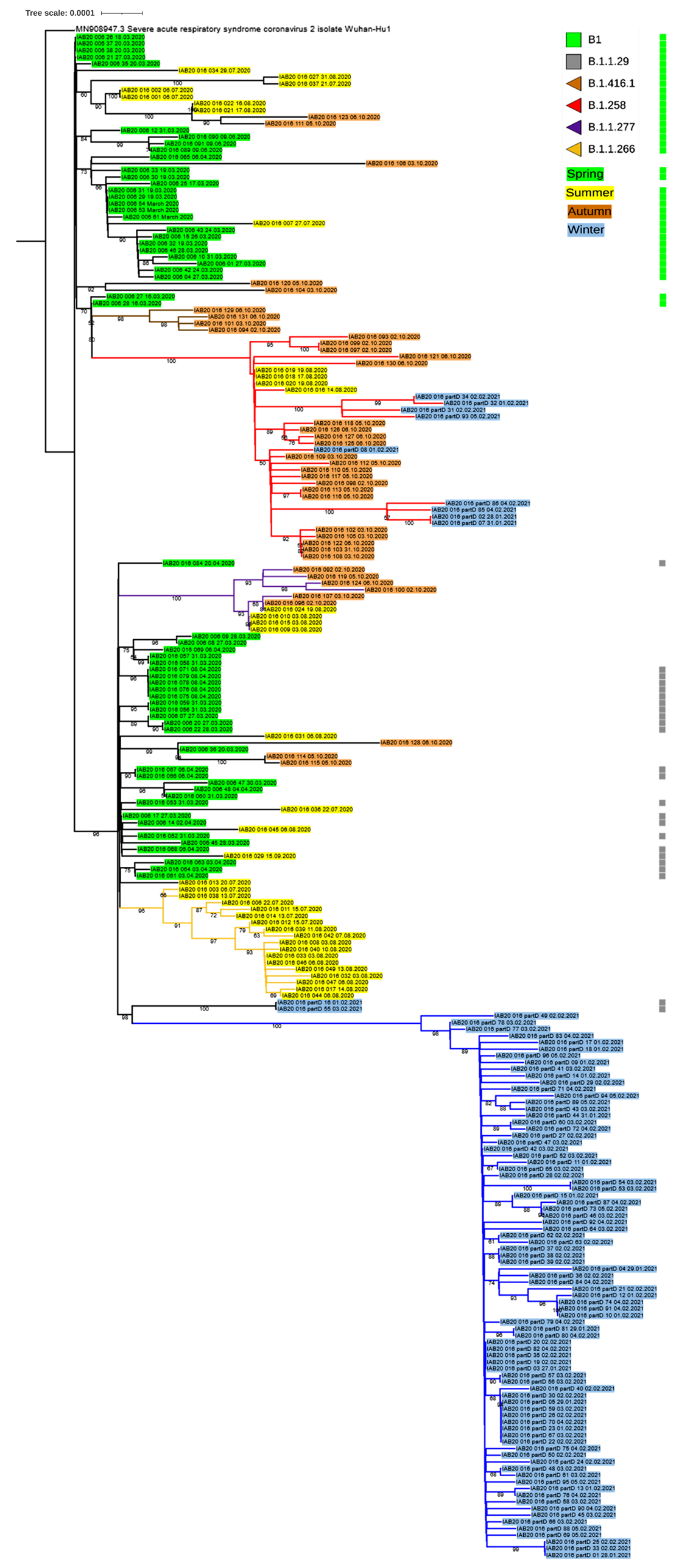

Supplement: Supplementary file 1 [file microorganisms-09-01671-s001.zip › RESUBMITTED Supplementary Materials MDPI REVISIONS/Figure_S1_PhylogeneticTree.tif]
